# Effects of Sublethal Concentrations of the Chitin Synthesis Inhibitor, Hexaflumuron, on the Development and Hemolymph Physiology of the Cutworm, *Spodoptera litura*

**DOI:** 10.1673/031.012.2701

**Published:** 2012-02-20

**Authors:** Qiqi Zhu, Yuan He, Jing Yao, Yinzhao Liu, Liming Tao, Qingchun Huang

**Affiliations:** Shanghai Key Laboratory of Chemical Biology, School of Pharmacy, East China University of Science and Technology, Shanghai, China

**Keywords:** sublethal effects, hexaflumuron, *Spodoptera litura*

## Abstract

The effects of sublethal concentrations 0.1, 0.5, and 1.2 µg mL^-1^of the chitin synthesis inhibitor, hexaflumuron, on larval growth and development, the count and proportion of hemocytes, and carbohydrate content (trehalose and glyceride) in hemolymph were investigated in the cutworm, *Spodoptera litura* (Fabricious) (Lepidoptera: Noctuidae). When 3^rd^instar larvae were subjected to the sublethal concentrations, there were dose-dependent effects on larval weight and length of each instar larvae, percent pupation and the duration of development. Most of the larvae died during the molting process at all concentrations. Few individuals from 0.5 and 1.2 µg mL ^-1^concentrations could develop to the 6^th^instar, while the pupae emerging from the 0.1 µg mL ^-1^concentrations did not exceed 16% of the number of the initial larvae. In 5^th^instar *S. litura,* the total number of hemocytes was significantly increased at 24 hours post—treatment, whereas the proliferation of hemocytes was inhibited, plasmatocyte pseudopodia contracted, and granulocyte expanded at 96 hours post—treatment. The increases of plasmatocyte count and the decreases of granulocyte count were dose—dependent. The longer treatment time of the sublethal concentrations increased the content of total carbohydrate and trehalose in hematoplasma, and was dose—dependent in hemocytes. The content of glyceride in hemolymph was significantly higher at 24 hours post—treatment, but gradually returned to normal levels at 96 hours post—treatment as compared with the control. The results suggested that sublethal concentrations of hexaflumuron reduced *S.* *litura* larval survival and interfered with hemolymph physiological balances.

## Introduction

The cutworm, *Spodoptera litura* (Fabricious) (Lepidoptera: Noctuidae), is an economically important and regular polyphagous pest, which seriously harms cabbage, soybeans, cotton, and other vegetables and cash crops in China (Xue et al. 2010). Benzoylphenylureas insecticides, a group of insect cuticular chitin synthesis inhibitors, are usually used to protect the miscellaneous vegetables from insect damage, and specifically act on the incorporation of N-acetyl glucosamine monomer into chitin in the integument, resulting in the formation of abnormal new cuticle and death of the insect (Nakagawa et al. 1993, 1994, 1996). Sulfonylurea receptor (SUR) in epidermal cells is its potential target (Abo-Elghar et al. 2004; Matsumura 2010).

The sustained effect of insecticides on insect physiology and behavior has been studied at lower doses that are not life endangering (Delpuech et al. 1998). Sublethal doses of insecticides may be potentially toxic to different instars and stages of insects through diverse effects such as interfering with the function of glutathione *S*-transferases, carboxylesterase, and other metabolic enzymes, or changing the behavior patterns associated with feeding, migration, reproduction, and/or the exchange of chemical information (Haynes 1988; Lee 2000). Buprofezin caused weight loss of *S. littoralis* and pyriproxyfen decreased body weight, extended the duration of larval and pupal development, and reduced the pupation of *S. littoralis* at doses without significant mortality (Nasr et al. 2010). Therefore, studies of sublethal effects of insecticides against insects can influence application of insecticides and potentially reduce negative environmental effects.

Insect growth regulators that govern an insect's maturation process usually provide long—lasting residual effects against insect pests. In this paper, the effect of hexaflumuron with sublethal concentrations on the development and hemolymph physiology of *S. litura* were investigated. The aims were to characterize the growth and development of *S. litura* larvae and the physiological adaptation of larval hemolymph composition through the sustained action of hexaflumuron at sublethal concentrations.

## Materials and Methods

### Insects

The common cutworm, *S. litura,* was reared continuously with fresh cabbage leaves, and the insects were laboratory—acclimated strains reared under controlled experimental conditions: 25 ± 1 ^°^C, 60–70% RH, and 16:8 L:D photoperiod. The insects were deprived of food for four hours prior to being used in activity bioassays.

### Chemicals

Hexaflumuron (97.5%) was supplied by Jiangsu Yangnong Chemical Group Co., Ltd. (www.yangnong.com.en). Stock solutions were prepared in N,N-dimethylformamide at a concentration of 10.0 mg mL^-1^, and diluted to the desired concentrations for usage with distilled water containing Triton X-100 (1.0 mmol L^-1^).

Phenylthiourea (Grade II, approx. 90%) was purchased from Sigma-Aldrich (www.sigmaaldrich.com). Anthrone, sulfuric acid, glucose, sulfosalicylic acid, trichloroacetic acid, triolein, acetyl acetone, sodium iodide, Triton X-100, and other chemicals were analytical reagents that were purchased from Shanghai Lingfeng Chemical Reagent Company.

### Experimental regime

Leaf—dip experiments were conducted as described previously (Huang et al. 2003). Fresh cut cabbage leaves were dipped in a diluted solution of hexaflumuron for 10 seconds, dried at room temperature, and then fed to the two—day—old second instar *S.* *litura* that had been deprived of food for four hours. Leaves treated with the solution without hexaflumuron served as controls. The bioassay was repeated three times with 10 larvae per replicate for each concentration. Mortality was monitored at 72 hours, and larvae that showed no responses to a needle probe were considered dead. The lethal concentration (LC_20_, LC_50_, and LC_70_) values of hexaflumuron were calculated with PoloPlus program (LeOra Software, version 1.0).

A similar technique was used to assess the sublethal effect of hexaflumuron at the dosage of LC_20_, LC_50_, and LC_70_ levels on the larval growth of 3^rd^ instar *S. litura.* With one larva per Petri dish (6.0 cm diameter), not less than 100 larvae were tested for each concentration and continuously fed the dipped leaves until the larvae metamorphosed to the pupae. The age, weight, and stem length of each surviving larva were determined at 24—hour intervals. The survival rate and the development duration of the larvae were also determined every 24 hours..

### Obtaining hemolymph and assay of hemolymph samples

The one—day—old 5^th^ instar *S. litura* that had been deprived of food for four hours were continuously fed the dipped leaves. At 24 hours and 96 hours post—treatment, hemolymph samples were obtained from chilled,surface—sterilized larvae. Larval hemolymph was drawn from the hemocoel by puncturing the propterothoracic membrane at the base of the coxa of the metathoracic leg, collected in an eppendorf tube containing phenylthiourea crystals, and used as hemolymph samples. 200 µL of hemolymph was centrifuged (300 g, 4 ^°^C) for 10 min. The supernatants were collected as hematoplasma samples. The pellets were resuspended in 200 µL of distilled water and sonicated with a tip sonifier for 15 seconds to serve as hemocyte samples.

To determine the total hemocyte count, 20 µL of the hemolymph was diluted 1:9 (v/v) in chilled saline (NaCl 7 g L^-1^ KC1 0.2 g L^-1^CaCl_2_0.2 g L^-1^, MgCl_2_0.1 g L^-1^, NaHCO_3_ 0.15 g L^-1^, NaH_2_PO_4_ 0.2 g L^-1^, Glucose 7.0 g L^-1^), and aliquots were transferred to a Neubauer hemocytometer. The cells were counted using a light microscope.

To determine the different type of hemocytes, hemolymph smears were made as described previously (Beetz et al. 2008). The hemocytes were identified using phase contrast microscopy and the morphological features were differentiated according to previous descriptions (Lavine et al. 2002; Ribeiro et al. 2006; Strand 2008).

To determine the content of carbohydrate, the anthrone colorimetric method was used as described by Leyva et al (2008) with minor modifications. 100 µL of samples were diluted 5 times with 10% sulfosalicylic acid to fully precipitate the protein and centrifuged (1000 × g) for 10 min. The supernatants were then collected. The content of carbohydrate was determined by using 50 µL of the supernatant of each sample with a standard prepared from 100 µg mL^-1^ glucose.

To determine the content of trehalose, the samples were determined with anthrone reagent as described by Michitsch et al. (2008). 100 µL of samples were mixed with 200 µL of 10% trichloroacetic acid. Following centrifugation (1,360 × g) for 5 min, 50 µL of the supernatants was transferred to a chilled tube and 2.5 mL of cold anthrone reagent was added and vigorously mixed in the ice bath. The content of trehalose was determined with the absorbance read at 620 nm and a standard prepared from 100 µg mL--^1^ trehalose.

To determine the content of glyceride, the samples were determined with acetylacetone reagent as described previously (Nanjing Agricultural University 1991). 100 µL of the samples were mixed with 3.0 mL of the extraction solvent (heptane: isopropyl alcohol 2:3:5 (v/v)) for 5 min by a vortex mixer, then 1.0 mL of 0.04 M sulfuric acid was added and mixed well. After standing by for 2 min, 1.0 mL of the supernatant was transferred to a glass tube, mixed with 1.5 mL saponification agent (KOH 0.5g, ddH_2_O 10 mL, isopropanol 100 mL) and incubated at 65 ^°^C for 5 min, then 1.0 mL of oxidation reagent (CH_3_COONH_4_ 7.7 g; CH_3_COOH 10 mL; INaO_4_ 0.lg; ddH_2_0 490 mL) and 1.0 mL of acetylacetone reagent (acetylacetone 0.4 mL, isopropyl alcohol 100.0 mL) were added. The mixture was continuously incubated at 65 ^°^C for 15 min. After cooling, the color was read in a spectrophotometer at 420 nm.

### Statistical analysis

Data on the growth parameters of each surviving larvae were determined. The quantitative estimations of the content of carbohydrate, trehalose, and glyceride were determined by three replicates and the % content relative to the control T/C × 100 calculated, where T and C represent the amounts in the treated larvae and in the control, respectively. All data were presented as means ± SE. Statistical significance was determined using the one—way analysis of variance (ANOVA) and separated by a least significant difference multiple range test, and a probability level *P* < 0.05 was considered statistically significant by SPSS version 13.0 (Hao et al. 2009).

## Results

### Sublethal effects of hexaflumuron on larval growth and development

**Figure 1.**
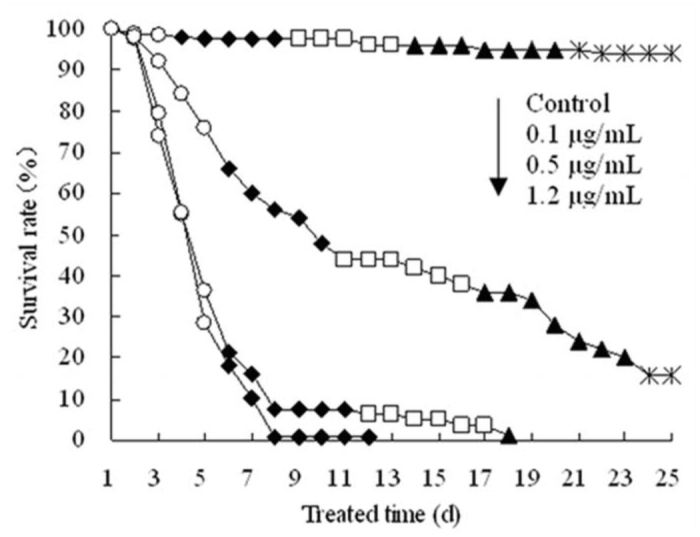
Survival regulation and subsequent development of 3^rd^ instar larvae after continuously fed with 0.1, 0.5, and 1.2 µg mL^-1^ hexaflumuron—dipped cabbage leaves. (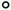 3^rd^ instar, 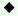
4^th^instar, 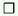
5^th^instar, 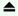
6^th^instar, * pupae) High quality figures are available online.

Regression equation of toxicity of hexaflumuron against the 2^nd^ instar *S. litura* at 72 hours was determined as y = 0.415 + 1.335x (χ^2^ = 0.968), and the lethal concentrations of LC_20_, LC_50_, and LC_70_ were estimated as 0.1 ± 0.014, 0.5 ± 0.063, and 1.2 ± 0.108 µg mL^-1^, respectively. After continuous feeding with treated leaves, sustained and cumulative sublethal toxicities of hexaflumuron were seen on the survival of 3^rd^ instar *S. litura* and their subsequent development. As shown in Figure 1, at the concentrations of 0.5 and 1.2 µg mL^-1^, the survival rate of 3^rd^ instar *S. litura* gradually decreased with the increase in treatment time. Most larvae died during the molting process after they developed to the 4^th^ instar, and a few individuals developed to the 6^th^ instar and then died, while most larvae fed with 0.1 µg mL^-1^ hexaflumuron were still alive with a survival rate of 16 percent by 24 days post—treatment when the surviving individuals developed into the pupae.

The sublethal effects of hexaflumuron on the developmental duration, larval weight, and stem length of *S. litura* larvae were further determined at various growth stages as shown in Table 1. The prolongation of development duration and the inhibition of body weight and stem length of the 3^rd^, 4^th^, and 5^th^ instar *S.* *litura* were increased with the sublethal concentrations of hexaflumuron. At 0.5 µg mL-^1^ concentration, the 5^th^ instar *S.* *litura* had significantly smaller stem length and lighter body weight, but survived for a longer development time before death as compared with the control. The 6^th^ instar *S. litura* survived at 0.1 µg mL-^1^ concentration and pupated earlier than the control larvae, but there were not obvious differences between the treated pupae and the control (*p* < 0.05, ANOVA).

### Sublethal effect of hexaflumuron on larval hemocytes

The changes of total hemocyte count in the 5^th^ instar *S. litura* were affected by continuous feeding with 0.1, 0.5, and 1.2 µg mL^-1^ hexaflumuron. As shown in Table 2, total hemocyte count significantly increased with sublethal concentrations of hexaflumuron 24 hours post—treatment, indicating that hemocytes in the larvae exhibited positive stress immunity in response to hexaflumuron. By 96 hours, total hemocyte count in the control increased to 12.6 × 10^3^ cells mL^-1^, while total hemocyte count in treatments of 0.5 and 1.2 µg mL^-1^ were still maintained the same level as the 24 hour post—treatment, indicating that the sublethal concentrations inhibited the normal proliferation of hemocytes at 96 hours post—treatment.

**Table 1.**
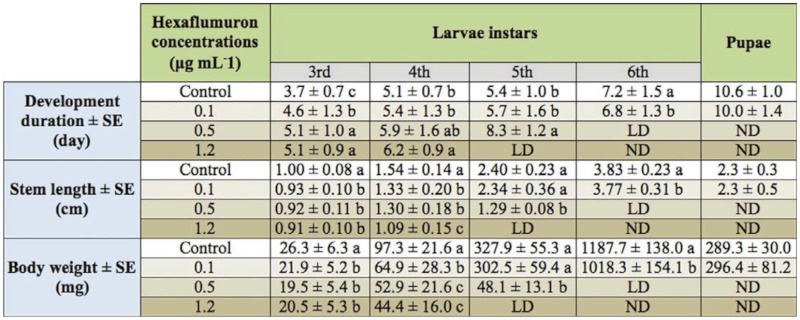
Effects of sublethal concentrations of hexaflumuron on the development duration, body weight, and stem length of *Spodoptera litura* larvae. Note: Means followed by the same letter in the same column are not significantly different (*p* < 0.05, LSD test). LD, larvae died; ND, not determined.

**Table 2.**
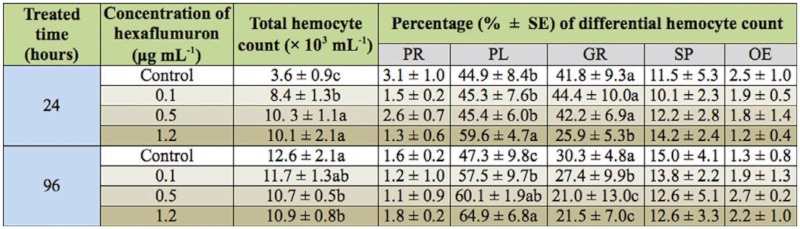
The counts of hemocytes in the 5^th^ instar larvae after continuous feeding with 0.1, 0.5, and 1.2 µg mL^-1^ hexaflumuron. Note: PR, prohemocyte; PL, plasmatocyte; GR, granulocyte; OE, oenocytoid; and SP, spherule cell. Means followed by the same letter in the same column are not significantly different (*p* < 0.05, LSD test).

**Figure 2.**
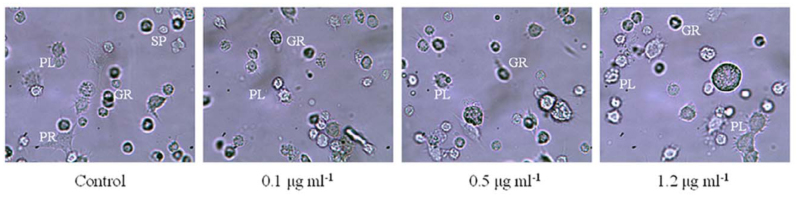
Morphological changes of hemocytes of *Spodoptera litura* after continuously fed with 0.1, 0.5, and 1.2 µg mL^-1^ hexaflumuron—dipped cabbage leaves for 96 hours (25Ox). High quality figures are available online.

Five different types of hemocytes in 5^th^ instar *S.* *litura* were identified as prohemocytes, plasmatocytes, granulocytes, oenocytoids, and spherule cells. Statistical data in Table 2 showed that the counts of plasmatocytes and granulocytes increased the most followed by the number of spherule cells. In the control,the number of plasmatocytes gradually increased along with the larval growth, while the number of granulocytes decreased simultaneously. Sublethal concentration of hexaflumuron seemed to play a strong facilitating role on promoting the increases of plasmatocytes and the decrease of granulocytes. Especially in the treatment of 1.2 µg mL^-1^, the percentages of the count of plasmatocytes and granulocytes were changed to 64.9% and 21.5%, respectively, showing significant difference relative to the control at 96 hours post—treatment (*p* < 0.05, ANOVA). However, few effects of the sublethal concentrations were revealed on the number of spherule cells, oenocytoids, and prohemocytes. Morphological changes of hemocytes of *S.* *litura* showed that 1.2 µg mL-^1^ hexaflumuron could cause plasmatocyte pseudopodia to contract and shorten, and granulocytes to swell and expand (Figure 2).

### Sublethal effect of hexaflumuron on carbohydrate level in larval hemolymph

**Figure 3.**
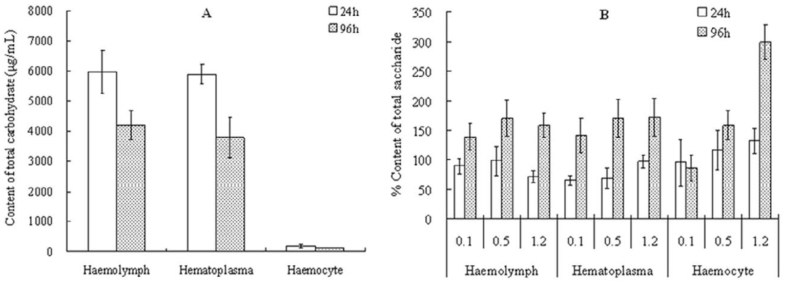
Sublethal effects of 0.1, 0.5, and 1.2 µg mL^-1^ hexaflumuron on the content of total carbohydrate in 5^th^ instar *Spodoptera litura* larvae. (A) Content of total carbohydrate in the control; (B) percent content of total carbohydrate in the treatments relative to the control. High quality figures are available online.

In the hemolymph extracted from the control larvae, only 2.5–4.4% of total carbohydrate was found in the hemocytes. As shown in Figure 3A, total carbohydrate in hemolymph, hematoplasma, and hemocytes decreased gradually in controls with the increasing age of the 5^th^ instar *S. litura.* In the treatments with 0.1, 0.5, and 1.2 µg mL-^1^ hexaflumuron, total carbohydrate slightly decreased in hematoplasma, but was significantly dose—dependently increased in hemocytes at 24 hours post—treatment (*p* < 0.05, ANOVA) (Figure 3B). Moreover, longer treatment time was shown to result in higher total carbohydrate in hematoplasma and hemocytes relative to the control. Exposure to 1.2 µg mL-^1^ hexaflumuron caused the total carbohydrate in hemocytes to increase to 3.0 times the control at 96 hours post—treatment.

### Sublethal effect of hexaflumuron on trehalose level in larval hemolymph

**Figure 4.**
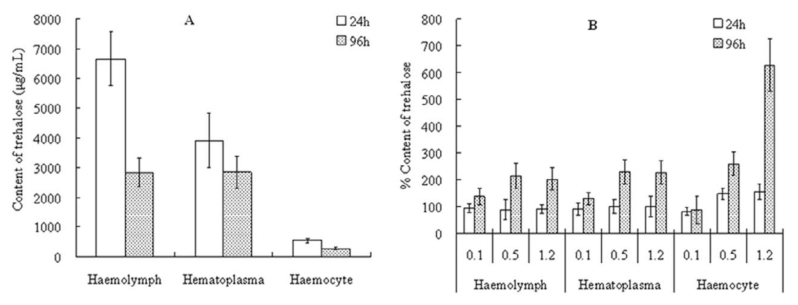
Sublethal effects of 0.1, 0.5, and 1.2 µg mL^-1^ hexaflumuron on the content of trehalose in 5^th^ instar *Spodoptera litura* larvae. (A) Content of trehalose in the control; (B) percent content of trehalose in the treatments relative to the control. High quality figures are available online.

Total trehalose in control hemocytes was 7.7– 9.3% in proportion with the total content in hemolymph that decreased gradually with the increasing age of the 5^th^ instar *S. litura* (Figure 4A). After exposure to sublethal hexaflumuron for 24 hours, the total trehalose in hemolymph and hematoplasma was not significantly different, but was significantly increased in hemocytes (*p* < 0.05, ANOVA). At 96 hours post—treatment, total trehalose in hemolymph and hematoplama was higher than in the control, and was significantly dose—dependently increased in hemocytes. Especially in the treatment with 0.5 and 1.2 µg mL-^1^ hexaflumuron, the total trehalose in hemocytes increased by 2.6- to 6.0-fold higher than the control, respectively (Figure 4B).

### Sublethal effect of hexaflumuron on glyceride level in larval hemolymph

**Figure 5.**
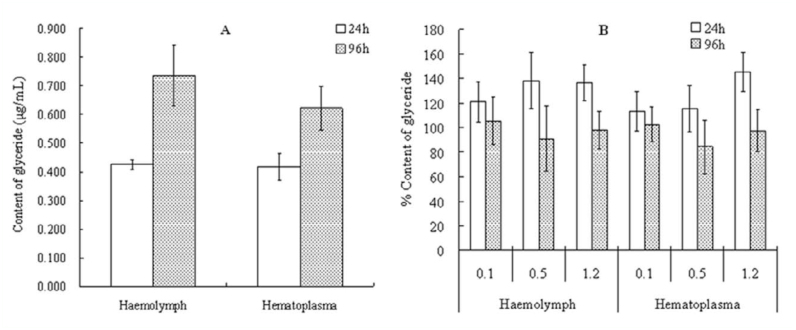
Sublethal effects of 0.1, 0.5, and 1.2 µg mL^-1^ hexaflumuron on the content of glyceride in 5^th^ instar *Spodoptera litura* larvae. (A) Content of glyceride in the control; (B) percent content of glyceride in the treatments relative to the control. High quality figures are available online.

Total glyceride in hemolymph and hematoplasma increased gradually with increasing age of the control 5^th^ instar *S. litura,* but was too low to be detected in hemocytes (Figure 5A). In the treatments of the sublethal concentrations of hexaflumuron (Figure 5B), the total glyceride in each treatment was significantly higher than in the control (*p* < 0.05, ANOVA), and the dosage of 1.2 µg mL-^1^ even induced 1.4-fold increases of the total glyceride in hematoplasma relative to the control at 24 hours post—treatment. However, total glyceride in each treatment was then decreased with the elongation of the treatment time, and gradually restored to the same level by 96 hours post—treatment as compared with the control (*p* < 0.05, ANOVA).

## Discussion

Sublethal insecticides had potential toxicity on the insect pests by interference effects on the feeding, odor dectection, courtship and mating behaviors, fecundity, and various metabolic enzymes (Delpuech et al. 1998; Haynes 1988; Lee 2000; Nasr et al. 2010). Low concentrations of chlorpyrifos could promote the growth of larvae, lead a significant increase to larval and pupal weight, and shorten the development duration of *S.* *litura* (Tang et al. 2009). Sublethal doses of pyrethroid insecticides interfered not only with larval development duration, the fecundity of female moth, and hatching rate of eggs of *Ostrinia furnucalis,* but also sex pheromone communication systems resulting in pheromone communication systems drift (Yang et al. 2003). Like other benzoylphenyl ureas, hexaflumuron altered cuticle composition, especially that of chitin, thereby affecting the firmness of the endocuticle (Grosscurt 1978). The reduced level of chitin in the cuticle seemed to result from inhibition of biochemical processes leading to chitin formation (Post et al. 1974; Hajjar and Casida 1979). Some of the reports indicated the possibility that benzoylphenyl ureas might affect the insect hormonal sites, thereby resulting in physiological disturbances such as inhibition of DNA synthesis (Soltani et al. 1984), altered carbohydrase and phenoloxidase activities (Ishaaya and Casida 1974; Ishaaya and Ascher 1977), or suppressed microsomal oxidase activity (Van Eck 1979). By continuously feeding on sublethal concentrations of hexaflumuron, our results showed that the mortality of *S. litura* larvae was dosage—dependently increased with increasing the treatment time. Most larvae died due to epidermal breakdown and the loss of body fluids at the molting stage. Exposure further caused the growth and development of the surviving larvae to be significantly inhibited, larval body weight and stem length to be reduced, and the development time to be lengthened, indicating that hexaflumuron could be accumulated in the larval body; thus, a cumulative toxicity against *S.* *litura* was exhibited.

Circulating hemocytes have important functions on the immune system, metabolism, and detoxification, and play a crucial role in the defense of xenobiotics or microbial infection. Sharma et al. (2008) showed that the number and proportion of different hemocytes were beneficial for insects to develop environmental fitness. In *S.* *littoralis* larvae, metyrapone caused the proportion of granulocytes to decrease while the proportion of plasmatocytes increased (Gelbič et al. 2005). In *S. litura* larvae, granulocytes and plasmatocytes were the most sensitive hemocytes to 0.15% azadirachtin, and the proportion of plasmatocytes decreased while the proportion of granulocytes increased. Other studies showed that sweet flag rhizome oil led to a decrease in the proportion of plasmatocytes and spherule cells while the proportion of granulocytes increased in *S. litura* larvae (Sharma et al. 2003, 2008). In *Agrotis ipsilon,* sublethal doses of dimilin decreased the total number of hemocytes, significantly increased the proportion of plasmatocytes, granulocytes, and spherule cells, and slightly decreased the proportion of prohemocyts (Nahla et al. 2009).

We found that exposure to sublethal concentrations of hexaflumuron 24 hours post—treatment resulted in a significant increase in the total hemocyte count of *S. litura,* followed by a decrease in the total hemocyte count level that was significantly lower than the control at 96 hours post-treatment. This result might be attributed to the release of hemocytes that adhered on surfaces within the hemocoel 24 hours post—treatment, and the inhibition of larval hematopoietic function and/or the cell proliferation after 96 hours post—treatment with sublethal hexaflumuron. Moreover, plasmatocytes and granulocytes in *S. litura* larvae were most sensitive to the sustained action of sublethal concentrations of hexaflumuron that resulted in an increase in the proportion of plasmatocytes and a decrease in the proportion of granulocytes. These data imply that sublethal hexaflumuron could strongly interfere with the differentiation of hemocytes, and thereby decrease the capability of larval immune defenses.

The content of carbohydrates and lipids in the hemolymph was an important indicator of the level of metabolism in insects, and a dynamic balance of the absorption, metabolism, and utilization by different tissues. Other studies showed that sublethal doses of fenitrothion and ethion continuously increased the content of trehalose and glucose, whereas pyriproxyfen at sublethal doses reduced glucose levels in haemolymph of *Bombyx mori* (Nath 2003; Etebari et al. 2007).

We found that carbohydrate and trehalose existed mainly in hematoplasma, and the amounts decreased gradually with the increasing age of *S. litura* larvae. However, the content of glyceride in hemolymph and hematoplasma increased simultaneously. Sublethal doses of hexaflumuron increased the content of carbohydrate and trehalose in hemolymph and hematoplasma. Especially in hemocytes, this increase was significantly dose—dependent and increased with treatment time. No significant difference in the content of glycerol in hemolymph and hematoplasma were found between the treatments and the control at 96 hours post—treatment. The content of carbohydrates and lipids in insect hemolymph is regulated by the adipokinetic hormone secreted by corpus cardiacum, and this hormone could increase the level of trehalose and reduce the level of lipid in hemolymph (Michitsch et al. 2008; Van der Horst et al. 1997). Whether sublethal concentrations of hexaflumuron affects the content of the carbohydrates and lipids by interfering with hormone balance is unknown.

The properties of sublethal effects of insecticides have attracted much attention of biologists and chemists. Sublethal concentrations of hexaflumuron not only significantly inhibited larval growth and extended the duration of development of *S. litura,* but also affected the metabolism of hemolymph carbohydrates and lipids, and changed the total number and the proportion of the circulating hemocytes, thereby affecting immune function and the survival of *S.* *litura.* Insecticides can also have sublethal effects on beneficial insects and may ultimately cause them to become less effective in parasitizing and preying on hosts (Amir et al. 2004). The residual activity of insecticides can enhance selection for insect resistance. Therefore, predicting the overall effects of insecticide use, including mortality and sublethal effects in insects, can facilitate the development of truly selective insecticides that can be employed in integrated pest management strategies. The results of evaluating the sublethal effect of hexaflumuron suggested that substantial physiological events in the life of *S. litura* larvae are involved in responding to the action of the insecticide.
